# Editorial: Ketone bodies: friend or foe?

**DOI:** 10.3389/fendo.2024.1400206

**Published:** 2024-03-22

**Authors:** Felix Sternberg, Mitsunori Nomura, Min Xie, Kalina Duszka

**Affiliations:** ^1^Department of Nutritional Sciences, Faculty of Life Sciences, University of Vienna, Vienna, Austria; ^2^Institute of Physiology, Pathophysiology and Biophysics, Department of Biomedical Sciences, University of Veterinary Medicine Vienna, Vienna, Austria; ^3^Buck Institute for Research on Aging, Novato, CA, United States; ^4^Department of Medicine, Division of Cardiovascular Disease, University of Alabama at Birmingham, Birmingham, AL, United States; ^5^Center of Translational Medicine, Warsaw University of Life Science, Warsaw, Poland

**Keywords:** betahydroxybutyrate (BHB), ketogenic diet (KD), adverse (side) effects, animal model, ketones, ketogenic diets, fatty acids (FA)

The therapeutic utilization of ketone bodies (KBs), induced through ketogenic diets (KDs)—high-fat, very low-carbohydrate regimens—has a history spanning over seven decades. This dietary approach has proven beneficial for individuals suffering from intractable epilepsy, Parkinson’s disease, diabetes, and cancer, owing to the significant enhancements in KB levels KDs facilitate. Ketone bodies, including acetoacetate, beta-hydroxybutyrate, and acetate, play a crucial role during states of starvation, low insulin signaling, and increased availability of fatty acids, thereby emerging as central to various therapeutic strategies. Despite their broad application, KDs association with increased health span and life span in mice (1, 2), anti-inflammatory effects on the gut (3), and the ongoing efforts to expand their use, especially in cardiology, recent studies have raised concerns about the potential off-target effects of KBs. Notably, preclinical animal model evidence points towards a diametrical impact of KBs on cardiac health (4–8), and their FA composition might also be crucial in a preclinical model of psoriasis (9). However, when carefully formulated, KDs have been evaluated as safe in children to manage epilepsy concerning cardiac parameters (10, 11). Similarly, another human KD study showed favorable outcomes for cardiovascular diseases (12), suggesting that the high ketogenic ratios and the limited variety in fat intake used in rodent preclinical models might not directly translate to human applications and a nuanced perspective is essential. This is in line with a meta-study indicating increased mortality when carbohydrates are replaced with animal-derived fats and proteins (13) underscoring the importance of careful consideration of the KD’s fat composition and duration, balancing the potential benefits against possible long-term health risks. Within this Research Topic, we collected one review article and 4 original research articles to set a starting point for future Research Topics.

In the concise review by Andersen et al., the authors elucidate the significant impact of dietary interventions, such as fasting and KD, on health, emphasizing the pivotal role of KBs—particularly beta-hydroxybutyrate (BHB)—in the regulation of stem cell biology. Beta-hydroxybutyrate is characterized not solely as an energy substrate during periods of fasting or adherence to low-carbohydrate diets but also as a critical signaling molecule that influences key stem cell functions, including quiescence, differentiation, and subsequent tissue regeneration. The review details how the response to KBs varies among different stem cell types, with muscle satellite cells entering a state of pronounced quiescence that limits regeneration, while intestinal stem cells see a delay in differentiation that promotes tissue regeneration due to KB supplementation.

Buchholz et al. present a pioneering phase I/II randomized clinical trial on the feasibility and efficacy of the Modified Atkins Diet (a less stringent form of KD) in managing mild cognitive impairment and early Alzheimer’s Disease. Their findings suggest that even trace amounts of ketones could enhance memory and vitality, marking a significant stride toward understanding dietary interventions in neurodegenerative conditions.

Dickens et al.’s study on adults with super-refractory status epilepticus undergoing a KD reveals intriguing correlations between increased KB production, alterations in lipid and inflammatory profiles, and clinical outcomes. This study not only adds to the growing body of evidence supporting KD’s benefits beyond epilepsy treatment but also opens new avenues for understanding the diet’s impact on inflammation and lipid metabolism.

Xie et al. delve into the complexities of hyperinsulinemia’s effects on the metabolic switch to KB utilization in proximal renal tubular epithelial cells, offering invaluable insights into potential therapeutic targets for renal protection under energy-deprived conditions. Their work highlights the delicate interplay between metabolic pathways in the face of hyperinsulinemia and starvation, pointing to the SIRT3/SMCT1 pathway as a critical regulator of KB absorption and utilization.

Finally, Diao et al.’s comprehensive analysis of diabetic kidney disease (DKD) underscores the pivotal role of KB metabolism in the disease’s progression. By identifying novel molecular signatures, their research paves the way for targeted therapeutic strategies that could mitigate DKD’s advancement, offering hope for those grappling with this condition.

As we conclude this editorial, it is clear that the contributions within this Research Topic not only illuminate the diverse roles of KB in health and disease but also underscore the burgeoning potential of KDs and KB supplementation in therapeutic settings. From regenerative medicine to neurodegenerative diseases, epilepsy, and beyond, the Research Topic navigates the complex landscape of KBs, offering fresh perspectives and beckoning further exploration into their therapeutic implications. This Research Topic serves as a beacon for future research, guiding us toward a more nuanced understanding of KBs and their place in medicine.

## Author contributions

FS: Conceptualization, Project administration, Supervision, Validation, Writing – original draft, Writing – review & editing. MN: Project administration, Supervision, Validation, Writing – original draft, Writing – review & editing. MX: Project administration, Supervision, Validation, Writing – original draft, Writing – review & editing. KD: Project administration, Supervision, Validation, Writing – original draft, Writing – review & editing.

## References

[1] Newman JC Covarrubias AJ Zhao M Yu X Gut P Ng CP. Ketogenic diet reduces midlife mortality and improves memory in aging mice. Cell Metab. (2017) 26:547–57.e8. doi: 10.1016/j.cmet.2017.08.004 28877458 PMC5605815

[2] Roberts MN Wallace MA Tomilov AA Zhou Z Marcotte GR Tran D. A ketogenic diet extends longevity and healthspan in adult mice. Cell Metab. (2018) 27:1156. doi: 10.1016/j.cmet.2018.04.005 PMC5957496 29719228

[3] Gregor A Huber L Auernigg-Haselmaier S Sternberg F Billerhart M Dunkel A. A comparison of the impact of restrictive diets on the gastrointestinal tract of mice. Nutrients. (2022) 14. doi: 10.3390/nu14153120 PMC9370610 35956298

[4] You Y Guo Y Jia P Zhuang B Cheng Y Deng H. Ketogenic diet aggravates cardiac remodeling in adult spontaneously hypertensive rats. Nutr Metab (Lond). (2020) 17:91. doi: 10.1186/s12986-020-00510-7 33117428 PMC7586698

[5] Chu Y Zhang C Xie M. Beta-hydroxybutyrate, friend or foe for stressed hearts. Front Aging. (2021) 2:681513. doi: 10.3389/fragi.2021.681513 35309549 PMC8932950

[6] Tao J Chen H Wang YJ Qiu JX Meng QQ Zou RJ. Ketogenic diet suppressed T-regulatory cells and promoted cardiac fibrosis via reducing mitochondria-associated membranes and inhibiting mitochondrial function. Oxid Med Cell Longev. (2021) 2021:5512322. doi: 10.1155/2021/5512322 33959215 PMC8075689

[7] Xu S Tao H Cao W Cao L Lin Y Zhao SM. Ketogenic diets inhibit mitochondrial biogenesis and induce cardiac fibrosis. Signal Transduct Target Ther. (2021) 6:54. doi: 10.1038/s41392-020-00411-4 33558457 PMC7870678

[8] Sternberg F Sternberg C Dunkel A Beikbaghban T Gregor A Szarzynski A. Ketogenic diets composed of long-chain and medium-chain fatty acids induce cardiac fibrosis in mice. Mol Metab. (2023) 72:101711. doi: 10.1016/j.molmet.2023.101711 36958422 PMC10122051

[9] Locker F Leitner J Aminzadeh-Gohari S Weber DD Sanio P Koller A. The influence of ketogenic diets on psoriasiform-like skin inflammation. J Invest Dermatol. (2020) 140:707–10.e7. doi: 10.1016/j.jid.2019.07.718 31630847

[10] Francois LL Manel V Rousselle C David M. Ketogenic diet as an alternative therapy for children with refractory epilepsy: about 29 children. Arch Pediatrie. (2003) 10:300–6. doi: 10.1016/S0929-693x(03)00030-7 12818749

[11] Ozdemir R Kucuk M Guzel O Karadeniz C Yilmaz U Mese T. Does ketogenic diet have any negative effect on cardiac systolic and diastolic functions in children with intractable epilepsy?: One-year follow-up results. Brain Dev. (2016) 38:842–7. doi: 10.1016/j.braindev.2016.03.009 27066714

[12] Sharman MJ Kraemer WJ Love DM Avery NG Gomez AL Scheett TP. A ketogenic diet favorably affects serum biomarkers for cardiovascular disease in normal-weight men. J Nutr. (2002) 132:1879–85. doi: 10.1093/jn/132.7.1879 12097663

[13] Seidelmann SB Claggett B Cheng S Henglin M Shah A Steffen LM. Dietary carbohydrate intake and mortality: a prospective cohort study and meta-analysis. Lancet Public Health. (2018) 3:e419–e28. doi: 10.1016/S2468-2667(18)30135-X PMC6339822 30122560

